# Utilization of Inferiorly Based Dermofat Flap in Breast Reconstruction after Simple Mastectomy due to Gigantomastia

**DOI:** 10.1155/2013/248969

**Published:** 2013-12-05

**Authors:** A. Bogdanov-Berezovsky, Y. Krieger, Y. Shoham, E. Silberstein

**Affiliations:** Department of Plastic and Reconstructive Surgery and Burn Unit, Soroka University Medical Center, Faculty of Health Sciences, Ben-Gurion University of the Negev, 84101 Beer-Sheva, Israel

## Abstract

Gigantomastia (GM) is a rare disabling condition characterized by excessive breast tissue growth. To date, there is no universal classification and definition of GM. At present, GM is determined as weight over 1.5 kg per breast (Dancey et al., 2008) or 3% or more of the patient's total body weight (Dafydd et al., 2011). The lack of generally acknowledged approach regarding GM is expressed by the different methods of its treatment ranging from hormonal prescription to mastectomy and subsequent complex breast reconstruction (Shoma et al., 2011). We describe a treatment approach, including simple mastectomy and immediate breast reconstruction by an inferiorly based dermofat flap with silicone implants and nipple grafting.

## 1. Materials and Methods

**Figure 1:**
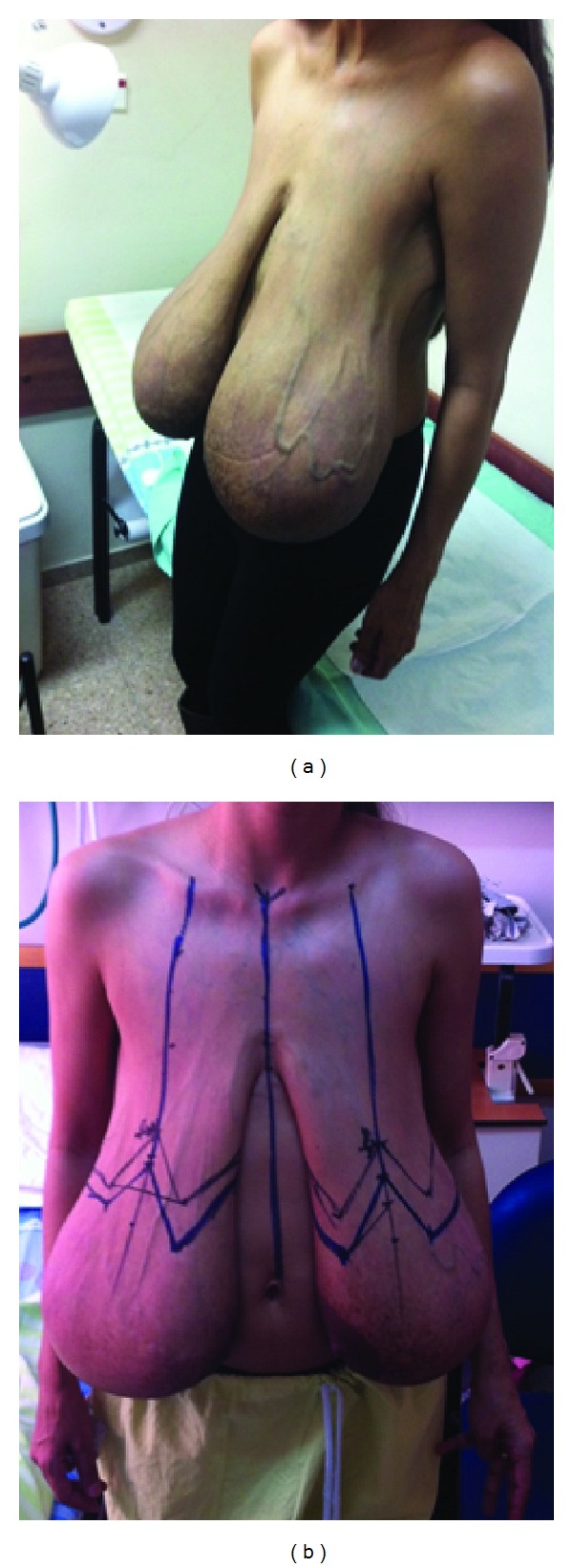
Pre-operative view. Extreme breast ptosis with medial rotation of nipple-areola complex.

*Case Report.* A 36-year-old otherwise healthy woman was initially referred to the breast clinic for the first time, complaining of large breasts, which had been steadily growing for 3 years since her first pregnancy. Her examination revealed large and ptotic breasts (DD cup bra) and reduction mammaplasty procedure was recommended to the patient; however, she preferred to postpone the surgery. Two years later, following the second delivery, she was referred to the clinic again, complaining of huge painful breasts, which were significantly affecting her quality of life, desperately seeking for medical care (Figure 1).

On the examination, huge ptotic breasts with the nipples at the pubis level were observed. The breasts were empty in the upper pole, with concentration of most of the breast tissue in the lower pole. Large subcutaneous veins were present on the chest wall and both breasts. Blood analyses and her hormonal profile were normal. After discussion with the patient, the decision to perform bilateral simple mastectomy with immediate breast reconstruction and free nipple grafting was made [1].

**Figure 2:**
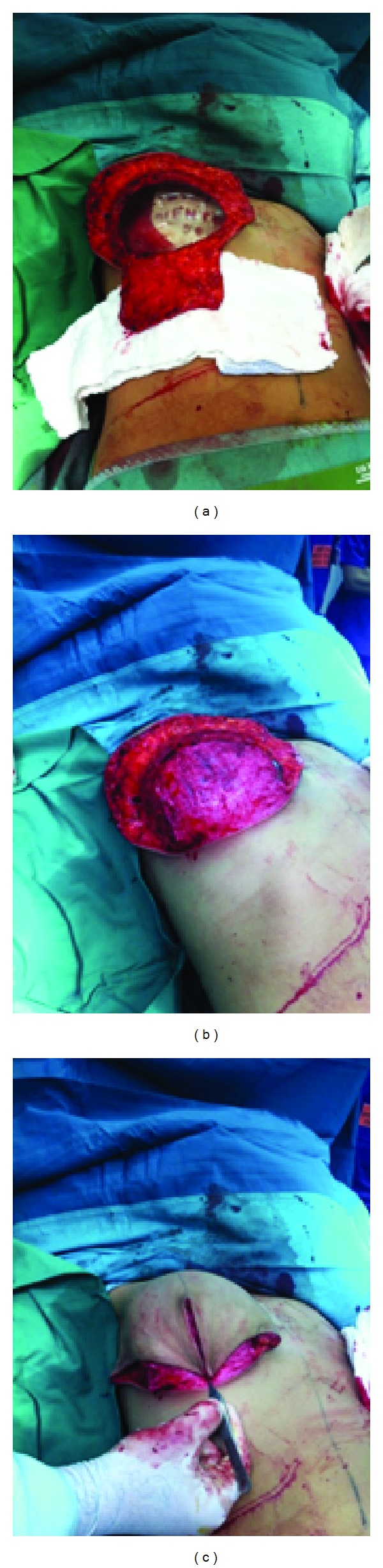
Subpectoral pocket was created; silicone sizer is in a place; IMF-based flap is ready for transfer and lower pole coverage (a); the flap covered the lower pole (b); and the skin envelope is redraped on the totally covered silicone implant (c).

**Figure 3:**
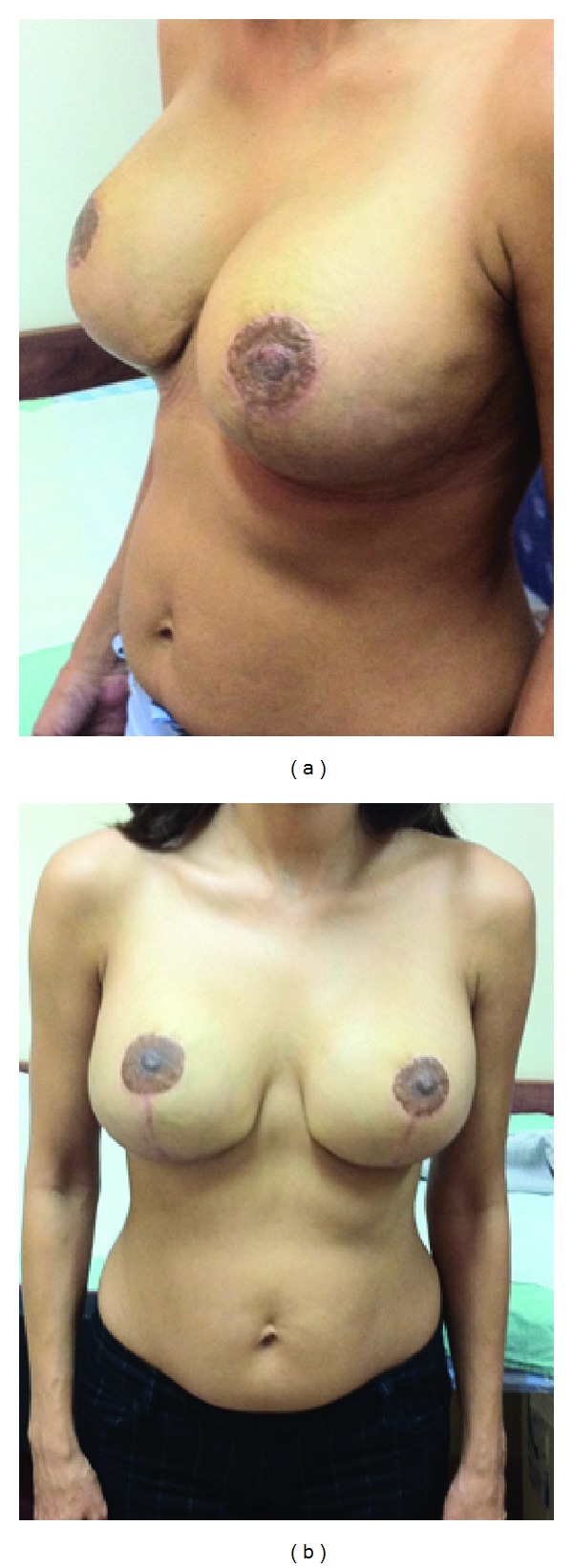
Postoperative result 6 months after the operation.

Under general anesthesia, skin incisions were made in the wise pattern fashion; the nipples were removed and preserved to be used as free nipple grafts. Preplanned dermofat flap, based on the inframammary fold (IMF), was designed and deepithelized. Simple subcutaneous mastectomy was performed through the wise pattern incisions. The weight of removed breasts was 4.9 kg (right breast: 2.6 and left: 2.3 kg) that compiled 9.3% of the total body weight (4.9/52.9 kg). Silicone implants (495 cc GCM Tall High Profile, Mentor Corp., Santa Barbara, CA, USA) were inserted into subpectoral pockets. The lower pole of the silicone implants was covered by the inferiorly based dermofat flap, which was sutured to the lower edge of the pectoral muscle. Total implant coverage was achieved, and the breast envelope was redraped on the implant. After the skin was sutured in the conventional wise breast reduction pattern (Figure 2), the operation was completed by free nipple grafting. Postoperative course was uneventful and a 6-month followup showed a good cosmetic postoperative result (Figure 3). The histological examination revealed fibrosis of breast tissue and sclerosing adenosis.

## 2. Discussion

Gigantomastia almost always represents a treatment dilemma that is still not well defined. The true incidence of GM is apparently underestimated and is approximately quoted as 1 : 100.000 [2, 3]. According to different publications, GM is considered when 0.8–1.5 kg breast tissue is removed per one breast during reduction mammoplasty [4]. A recent publication redefined GM as excess breast tissue that contributes 3% or more of the patient's total body weight [5]. The 4.9 kg weight of removed breast tissue from our patient is far from the highest records reported; however, it still represents 9.3% of her total body weight.

Our case raised a number of questions: what is the most appropriate type of surgery for this patient (breast reduction versus mastectomy)? What reconstructive method should be implemented for the patient? What size of breast implant should be used in this case? How to provide proper coverage for the implant during surgery (local tissues, tissue substitutes, or regional flap)?

The decision to perform a mastectomy was mostly based on the size of breasts, the probability of recurrent breast tissue growth, the patient's wish, and the possibility to create aesthetically acceptable and symmetrical breasts.

The silicone-based reconstruction was considered as the most suitable treatment for the patient according to her physique. The huge amount of redundant postmastectomy skin was utilized as an inferiorly based large dermofat flap for total coverage of the large silicone implant. The dermofat flap was considered by us as superior to any kind of tissue substitute that could be used for implant coverage. The dermofat flap is a simple, reliable, and reproducible flap, which can be used for total coverage of breast implants in medium-sized or large breasts.
